# Lessons from the INSEMA trial: is sentinel lymph node biopsy in early-stage breast cancer stepping off the stage?

**DOI:** 10.3389/fonc.2025.1564689

**Published:** 2025-04-09

**Authors:** Xiaoyan Chen, Jiapeng Xue, Zhi Li

**Affiliations:** ^1^ Department of Physical Therapy, Taihe Hospital, Hubei University of Medicine, Shiyan, China; ^2^ Medical College of Nanchang Institute of Technology, Nanchang, Jiangxi, China; ^3^ Department of General Surgery, Taihe Hospital, Hubei University of Medicine, Shiyan, China; ^4^ Interventional Cancer Institute of Chinese Integrative Medicine, Putuo Hospital, Shanghai University of Traditional Chinese Medicine, Shanghai, China

**Keywords:** breast cancer, sentinel lymph node biopsy (SLNB), axillary lymph node dissection (ALND), clinical benefits, adjuvant treatment of melanoma

## Key evidence and findings

The publication of the INSEMA trial results in *The New England Journal of Medicine* on December 12, 2024 (5), marks a pivotal moment in the treatment paradigm for early-stage breast cancer. Coupled with its presentation at the San Antonio Breast Cancer Symposium, this groundbreaking study challenges the traditional necessity of SLNB in low-risk early breast cancer, further reinforcing the trend toward de-escalation of axillary surgery.

The INSEMA trial enrolled over 5,500 clinically node-negative invasive breast cancer patients, predominantly hormone receptor-positive and HER2-negative. The results demonstrated that omitting SLNB did not negatively impact invasive disease-free survival (IDFS) rates, which were similar to those in the SLNB group, and the axillary recurrence rate was below 1% (1.0% in the no surgery-group vs. 0.3% in the SLNB-arm). These findings suggest that, in certain low-risk patients, omitting SLNB does not compromise treatment efficacy, thus supporting the safety and effectiveness of SLNB omission. These results align with those of the earlier SOUND trial (6), which focused on patients with tumors ≤ 2 cm (T1 tumors), further reinforcing the feasibility of omitting SLNB. The SOUND trial also showed that omitting SLNB did not result in significant clinical deterioration, as there were no differences in distant disease-free survival between the treatment arms. Together, these studies provide strong evidence for the omission of SLNB in breast cancer treatment, particularly for low-risk populations.

However, the results of the INSEMA trial are not without controversy, and the study highlights several areas that require further exploration. While the recurrence risk is low for T1 tumors, the safety of omitting SLNB for T2 tumors (tumor size >2 cm and ≤5 cm) has not been fully validated. In the INSEMA cohort, approximately 20.8% of clinical T2 patients had macrometastatic nodal disease, suggesting that omitting SLNB in this subgroup could potentially increase the risk of axillary recurrence. Therefore, more data are needed to guide clinical practice regarding SLNB omission in T2 tumor patients. Additionally, high-grade tumors and features such as lymphovascular invasion, which are typically associated with poorer prognosis, were underrepresented in the INSEMA trial. This calls for caution when applying the findings of this study to high-risk populations, as it may not be appropriate to generalize the SLNB omission strategy to all patients. This also underscores the need for future clinical research to consider tumor biological characteristics and patient risk assessment in order to more accurately define the indications for SLNB omission. Moreover, most patients presented with low-risk breast carcinoma, with a small number of high-risk cases (3.6% G3 tumors and patients with lymphangioinvasion), though their impact was not analyzed in detail.

One limitation of this study is the exclusion of patients with higher-risk tumors, such as HER2-positive or triple-negative breast cancer. These patients were not enrolled because they are typically candidates for neoadjuvant systemic therapy, which could influence nodal status and treatment decisions. Additionally, during the recruitment period, multigene signatures such as Oncotype DX and MammaPrint were not widely available. As a result, nodal status remained a key factor in determining the use of chemotherapy, particularly for G3 tumors. This may have impacted the generalizability of INSEMA findings, as treatment decisions in current clinical practice increasingly rely on molecular profiling rather than nodal involvement alone. Future studies incorporating modern genomic assays and including a broader range of tumor subtypes are necessary to further refine patient selection criteria for omitting SLNB.

## Clinical benefits of omitting SLNB

SLNB, originally a minimally invasive alternative to ALND, provides important staging information but does not offer therapeutic benefits (1–3). In this context, the INSEMA trial, alongside the earlier SOUND study, explored a critical question: can SLNB be safely omitted in carefully selected patients, especially during breast-conserving surgery? Omitting SLNB offers significant clinical benefits in breast cancer treatment, particularly in reducing surgical complications (4, 5). Although SLNB has a lower complication rate compared to traditional ALND, it still carries potential risks such as lymphedema, sensory loss, and limited arm mobility. For low-risk patients, especially those in carefully selected cohorts, omitting SLNB effectively improved patient-reported quality of life, particularly by reducing arm symptoms such as pain, swelling, and impaired mobility (7).

Furthermore, avoiding SLNB can also alleviate psychological burdens for patients. For many, the physical trauma and postoperative recovery time associated with lymph node dissection and biopsy are considerable factors (8). By omitting SLNB, patients experience smoother recovery, reduced postoperative discomfort, and shorter hospital stays. Therefore, omitting SLNB not only physically lightens the patient’s burden but also reduces psychological and social stress for breast cancer patients. Additionally, omitting SLNB aligns with the concept of personalized treatment. As precision medicine advances, more breast cancer treatment decisions are based on the tumor’s molecular characteristics and the patient’s overall health, rather than solely on traditional anatomical staging (9). This means that tumor biology and prognostic factors may, in some cases, be more decisive in determining treatment strategies than lymph node status. For high-risk groups, while omitting SLNB may not be feasible, in low-risk patients, accurate tumor staging and biomarker use can ensure the safety and efficacy of treatment.

However, omitting SLNB may lead to an increased reliance on endocrine therapy in more patients, as treatment decisions shift away from nodal status. While endocrine therapy is effective, it is not without side effects, including fatigue, osteoporosis, and thromboembolic events. This could negatively impact patients’ quality of life and should be carefully considered when weighing the benefits of SLNB omission.

## Impact on adjuvant treatment decisions

While SLNB provides important clinical information for breast cancer staging, its impact on adjuvant treatment decisions has gradually been overshadowed by other factors (10). With the widespread use of new targeted therapies and immunotherapies, breast cancer treatment no longer solely relies on traditional lymph node staging. Tumor-specific characteristics, such as HER2 status, hormone receptor expression, and tumor mutation profiles, have become key determinants of treatment plans. These therapies have ushered in a new era for breast cancer treatment, where molecular subtyping, gene mutations, and drug responses play a crucial role in decision-making, reducing reliance on the status of axillary lymph node metastasis.

For some low-risk patients, especially those with hormone receptor-positive, HER2-negative breast cancer, omitting SLNB does not compromise treatment efficacy but can avoid unnecessary surgical interventions. However, the parallel de-escalation of axillary surgery and radiotherapy remains an ongoing challenge. Currently, data supporting partial breast irradiation alone or breast-conserving surgery without postoperative radiotherapy are limited to proven sentinel node-negative patients (11). Therefore, studies recruiting clinically node-negative patients without SLNB are necessary to explore further de-escalation of postoperative radiotherapy. Limited data from the SOUND trial (6) indicate that approximately 10% of patients in both arms received partial breast irradiation alone, suggesting the need for further investigation into this approach. As breast cancer treatment becomes increasingly individualized, the focus of adjuvant therapy has shifted towards optimizing efficacy while minimizing side effects (12). For low-risk patients, effective disease control may still be achievable through appropriate endocrine therapy, targeted therapies, and radiotherapy, even without SLNB. Therefore, omitting SLNB can alleviate the treatment burden without compromising therapeutic outcomes, but careful consideration of radiotherapy strategies is essential.

## Future research directions

The INSEMA and SOUND trials offer new perspectives on breast cancer treatment, indicating that SLNB can be omitted in low-risk early-stage breast cancer patients undergoing primary breast-conserving therapy. However, it is important to note that these findings are limited to this specific patient group. Currently, no data are available regarding the omission of SLNB during primary mastectomy or after neoadjuvant systemic therapy. While these results provide clearer guidance for future treatment decisions, they also raise many questions that warrant further exploration. Future research should focus on precisely defining which patient populations are most suitable for omitting SLNB, particularly for T2 tumors and high-risk patients. Balancing the benefits and potential risks of omitting SLNB in individualized treatment strategies remains an urgent challenge. Moreover, as our understanding of breast cancer biology deepens, the role of the tumor microenvironment and immune system is receiving increasing attention. The immune microenvironment of breast cancer has a significant impact on tumor invasion, metastasis, and recurrence. Therefore, future studies may focus not only on the status of lymph node metastasis but also on tumor immune evasion mechanisms and the application of immunotherapy. The combination of immunotherapy and targeted therapies could further expand the use of SLNB omission strategies in specific patient groups. Additionally, collaboration within multidisciplinary teams will play a critical role in this field. Breast cancer treatment decisions are not solely made by surgeons but require the collective evaluation of experts from radiology, pathology, endocrinology, oncology, and other specialties. Multidisciplinary teams can help identify patients who are suitable for SLNB omission, considering tumor type, molecular characteristics, prognostic factors, and other variables to formulate the most optimal treatment plan.

## Conclusion

The INSEMA trial demonstrated that SLNB can be safely omitted in low-risk early-stage breast cancer patients, reducing unnecessary surgical interventions and alleviating both physical and psychological burdens for patients. This finding further advances the move toward personalized and precision-based breast cancer treatment. As new therapeutic methods emerge, the role of tumor staging is increasingly being replaced by tumor biology characteristics. The use of SLNB omission in specific patient populations will become an important trend in future breast cancer treatment. However, this change requires more detailed research and clinical validation to ensure optimal treatment outcomes across diverse patient groups.
